# Clinical Instruction

**Published:** 1857-10

**Authors:** 


*Clinical Instruction.*

Speaking of this subject in connection with Medical Colleges, the editor of the *Southern Journal of Medical and Physical Sciences*, in his September number, has the following paragraph:

"What are the opportunities for acquiring *Clinical* instruction, for, as lightly as some may speak of clinical lectures, they are essential to the making of the physician?"

"In this respect, the *New Orleans School of Medicine*, and *University of Michigan are superior to all other schools.*"

Alluding to the same subject, the *Medical Independent* for October, published at Detroit, has the following:

"The Clinical course in the *University* (*of Michigan*), established by the Regents at their spring meeting, commenced on the 26th of June, and closed on the 27th of August; consequently it had a duration of *two months.* The number of students in attendance was *nine.* Of these, seven remained through the whole two months; one, a graduate, left at the end of the first month; and one, a novice, was admitted at the middle of the term."

Such is the Clinical Course which the editor of the *Southern Journal* calls "superior to all other schools."

Verily! distance lends enchantment, &c.

